# Engaging patients in de-implementation interventions to reduce low-value clinical care: a systematic review and meta-analysis

**DOI:** 10.1186/s12916-020-01567-0

**Published:** 2020-05-08

**Authors:** Emma E. Sypes, Chloe de Grood, Liam Whalen-Browne, Fiona M. Clement, Jeanna Parsons Leigh, Daniel J. Niven, Henry T. Stelfox

**Affiliations:** 1 Department of Community Health Sciences, Cumming School of Medicine, University of Calgary, Calgary, Canada; 2 Department of Critical Care Medicine, Cumming School of Medicine, University of Calgary and Alberta Health Services, Calgary, Canada; 3 O’Brien Institute of Public Health, Cumming School of Medicine, University of Calgary, Calgary, Canada; 4 School of Health Administration, Faculty of Health, Dalhousie University, Halifax, Canada

**Keywords:** Low-value care, De-adoption, Patient engagement, Choosing wisely

## Abstract

**Background:**

Many decisions regarding health resource utilization flow through the patient-clinician interaction. Thus, it represents a place where de-implementation interventions may have considerable effect on reducing the use of clinical interventions that lack efficacy, have risks that outweigh benefits, or are not cost-effective (i.e., low-value care). The objective of this systematic review with meta-analysis was to determine the effect of de-implementation interventions that engage patients within the patient-clinician interaction on use of low-value care.

**Methods:**

MEDLINE, EMBASE, and CINAHL were searched from inception to November 2019. Gray literature was searched using the CADTH tool. Studies were screened independently by two reviewers and were included if they (1) described an intervention that engaged patients in an initiative to reduce low-value care, (2) reported the use of low-value care with and without the intervention, and (3) were randomized clinical trials (RCTs) or quasi-experimental designs. Studies describing interventions solely focused on clinicians or published in a language other than English were excluded. Data was extracted independently in duplicate and pertained to the low-value clinical intervention of interest, components of the strategy for patient engagement, and study outcomes. Quality of included studies was assessed using the Cochrane Risk of Bias tool for RCTs and a modified Downs and Black checklist for quasi-experimental studies. Random effects meta-analysis (reported as risk ratio, RR) was used to examine the effect of de-implementation interventions on the use of low-value care.

**Results:**

From 6736 unique citations, 9 RCTs and 13 quasi-experimental studies were included in the systematic review. Studies mostly originated from the USA (*n* = 13, 59%), targeted treatments (*n* = 17, 77%), and took place in primary care (*n* = 10, 45%). The most common intervention was patient-oriented educational material (*n* = 18, 82%), followed by tools for shared decision-making (*n* = 5, 23%). Random effects meta-analysis demonstrated that de-implementation interventions that engage patients within the patient-clinician interaction led to a significant reduction in low-value care in both RCTs (RR 0.74; 95% CI 0.66–0.84) and quasi-experimental studies (RR 0.61; 95% CI 0.43–0.87). There was significant inter-study heterogeneity; however, intervention effects were consistent across subgroups defined by low-value practice and patient-engagement strategy.

**Conclusions:**

De-implementation interventions that engage patients within the patient-clinician interaction through patient-targeted educational materials or shared decision-making tools are effective in decreasing the use of low-value care. Clinicians and policymakers should consider engaging patients within initiatives that seek to reduce low-value care.

**Registration:**

Open Science Framework (https://osf.io/6fsxm)

## Background

Clinical interventions that lack efficacy, have risks that outweigh benefits, or are not cost-effective constitute low-value care [1]. In the USA, overuse of low-value practices is estimated to cost upwards of 100 billion dollars annually [2] and is associated with adverse events, poor patient outcomes, and downstream use of healthcare resources [3, 4]. Reducing low-value care is therefore imperative for high-quality, sustainable healthcare. Researchers, governments, and public campaigns have commanded attention about low-value care by classifying hundreds of tests and treatments as low-value [5–8]. Yet, this increased awareness about low-value care has not translated into a reduction in use [9–11]. The use of low-value care may be reduced or stopped through de-implementation, which is defined as a planned process that uses targeted strategies such as education, incentives, or audit and feedback [12]. There is a need to understand how to achieve meaningful reduction in low-value care through comprehensive de-implementation interventions that acknowledge the complexity of this issue and appropriately engage researchers, decision-makers, clinicians, and patients.

Patients are directly involved in and impacted by low-value care and may play a pivotal role in solutions to reduce its use [13, 14]. However, the potential effects of patient engagement in de-implementation initiatives are complex. On the one hand, clinicians often cite patient demand for tests and treatments as a barrier to reducing low-value care [15, 16], while on the other hand, patients may experience mental and/or physical harm from unnecessary tests and treatments [4]. Making this question even more complex is the fact that inclusion of patients within initiatives to reduce low-value care may impute a mistrust within the patient-clinician interaction and create a false sense of the ubiquity of low-value care within the practice of medicine, while this is indeed not the case [2]. So, while it is assumed that patients should be engaged in any initiative that seeks to increase or decrease the use of clinical care, from the perspective of de-implementing low-value care, the risk/benefit ratio of such a patient-engagement strategy is not clearly defined, and a more thorough understanding is warranted before such a blanket recommendation. In a systematic review of interventions to reduce low-value care conducted in 2017, 26 of 108 included studies engaged patients within de-implementation interventions in some capacity (e.g., patient cost sharing, provider report cards) [17]. While the authors summarized the effects of patient engagement as “positive” through a narrative synthesis, the effect of such patient engagement was not quantified through meta-analysis, making it difficult to understand the true magnitude of effect and how it compares to interventions that focus on clinicians. The full extent of the impact of patient engagement in de-implementation initiatives remains unclear. In this study, we conducted a systematic review with meta-analysis to determine the effects of de-implementation interventions that engage patients within the patient-clinician interaction and quantify the impact of this patient involvement on the use of low-value care.

## Methods

### Protocol and guidance

This systematic review and meta-analysis is a follow-up to a scoping review that mapped the literature exploring the public’s role in reducing low-value care [18], the protocol for which was registered with Open Science Framework (https://osf.io/6fsxm). Methodology was guided by the Joanna Briggs Institute Reviewer’s Manual [19], and the reporting was guided by the Preferred Reporting Items for Systematic Reviews and Meta-Analyses (PRISMA) checklist [20].

### Search strategy and data sources

We searched MEDLINE, EMBASE, and CINAHL from inception to June 28, 2018, and the gray literature using the Canadian Agency for Drugs and Technologies in Health (CADTH) Grey Literature Search Tool [21]. The search strategy was developed in consultation with a medical librarian and peer reviewed by a second medical librarian using the Peer Review of Electronic Search Strategies (PRESS) Checklist [22]. The search strategy was initially developed in MEDLINE (Ovid) (Table 1) and subsequently translated for EMBASE and CINAHL databases with the help of a medical librarian. Our search terms included keywords and synonyms pertinent to three main concepts: (1) low-value care (e.g., overuse, de-implementation), (2) patients (e.g., consumers, patients), and (3) patient involvement (e.g., patient participation). Searches were limited to the English language as terminology regarding low-value care (e.g., Choosing Wisely, low-value, overuse, etc.) is unique to the English language and may not translate well across languages. Reference lists of included studies were hand-searched to identify additional citations, and suggestions were provided from experts in the field.

**Table 1:** MEDLINE (Ovid) search strategy

Line number	Search terms
1	health services misuse/or medical overuse/
2	Unnecessary Procedures/
3	((misuse* or overuse* or unnecessary or ineffective or overtreat* or overdiagnos* or overutilis* or overutiliz* or low value or waste*) adj5 (health or healthcare or care or procedure* or intervention* or test* or treatment*)).tw,kf.
4	((abandon* or contradict* or refute* or refuting or reassess* or re-assess* or obsole* or revers* or delist* or de-list* or disinvest* or dis-invest* or discontinu* or dis-continu* or decommission* or de-commission* or deadopt* or de-adopt* or de-implement* or deimplement*) adj5 (medical or health or healthcare or policy or procedure* or intervention*)).tw,kf.
5	1 or 2 or 3 or 4
6	patient participation/or community participation/
7	patient satisfaction/or patient preference/
8	((patient* or family* or families or public or citizen* or consumer*) adj5 (perception* or engag* or involv* or participat* or decision* or interaction* or role* or aware* or conversation* or responsibilit* or discuss*)).tw,kf.
9	6 or 7 or 8
10	5 and 9
11	choosing wisely.mp.
12	10 or 11
13	Limit 12 to English language

### Article eligibility and selection

Detailed inclusion and exclusion criteria are presented in Table 2. We used Elshaug’s definition of low-value care [1], which was operationalized to include clinical interventions (tests or treatments) that lack efficacy, have risks that outweigh benefits, or are not cost-effective. Citations were screened for inclusion in two phases. Prior to screening, the citation screening form was pilot tested using a random sample of 50 citations and refined until agreement was consistent (*k* > 0.8). In phase 1, two investigators (EES and CD) screened citations by title and abstract to determine potential eligibility. Potentially relevant citations entered into phase 2 screening where two investigators (EES and LWB) screened full-text versions of each citation to determine eligibility for inclusion. The kappa statistic was used to quantify agreement throughout screening [23].

**Table 2:** Inclusion and exclusion criteria

Inclusion criteria	Exclusion criteria
**Systematic review**	
Written in English	Reported an intervention to reduce low-value care that solely targeted clinicians
Described an intervention that engaged patients in their aim to reduce the use of low-value care*	Low-value practice of interest was not a medical test or treatment (e.g., bed rest, use of physical restraints)
Used experimental (e.g., randomized clinical trial) or quasi-experimental (e.g., controlled before-and-after study) study designs	
Reported the use of low-value care with or without the intervention	
**Meta-analysis**	
Measured the use of low-value care as the proportion of patients that received the low-value practice with and without exposure to the de-implementation intervention	

*Low-value care was defined as a clinical intervention that lacks efficacy, has risks that outweigh benefits, or is not cost-effective [1]

### Data extraction and risk of bias assessment

Data extraction was conducted in duplicate by three investigators (EES, CD, LWB) in DistillerSR (Evidence Partners, Ottawa, Canada). Our data extraction form was pilot tested using six randomly selected citations. Extracted data pertained to study characteristics (e.g., study design, country of origin), characteristics of the low-value intervention (e.g., test or treatment), components of the intervention (e.g., strategy for patient engagement, clinical setting), and outcomes (e.g., proportion of patients receiving low-value care).

Quality assessment was conducted in duplicate by two investigators (EES and LWB) using the Cochrane Risk of Bias Tool [24] for the RCTs and a modified Downs and Black checklist for the quasi-experimental studies [25]. For RCTs, summary assessments did not consider the “Performance Bias” domain, as participants were unable to be blinded due to the behavioral nature of the interventions. The Downs and Black checklist was modified by removing questions pertaining to randomization and control groups as necessary. Question 27 originally had six scoring options based on the percent change a sample was powered to detect, but was modified to “Did the authors conduct a power calculation? 1 = Yes, 0 = No” for simplicity. Due to these modifications, the checklist was scored out of 24, or 25 depending on the design of the study. Percent of the total possible score was calculated for each study to facilitate between-study comparisons. Studies were classified by three categories, which were determined by calculating the median percentage score and assigning scores below and above the median to the “lower quality” and “higher quality” categories, respectively. “Average quality” studies had overall percentage scores equivalent to the median.

### Data synthesis and analysis

The primary outcome was the proportion of patients who received a low-value clinical intervention. Individual study estimates of the primary outcome were pooled using the random effects model of DerSimonian and Laird [26] and reported as a risk ratio (RR). Publication bias was assessed using a funnel plot and Egger’s test. Heterogeneity was assessed using the *I*^2^ statistic [27] and Cochrane *Q* test. Explanations for heterogeneity were sought through stratified analyses and meta-regression. Pre-specified subgroups for stratified analysis included study design (RCTs vs. non-randomized), type of low-value care (test vs. treatment), and risk of bias (low vs. high/unclear). Meta-analyses were conducted using the metan package in Stata (version 14, StataCorp, TX, USA), and statistical significance was set as *P* < 0.05.

## Results

### Study selection

**Fig. 1:**
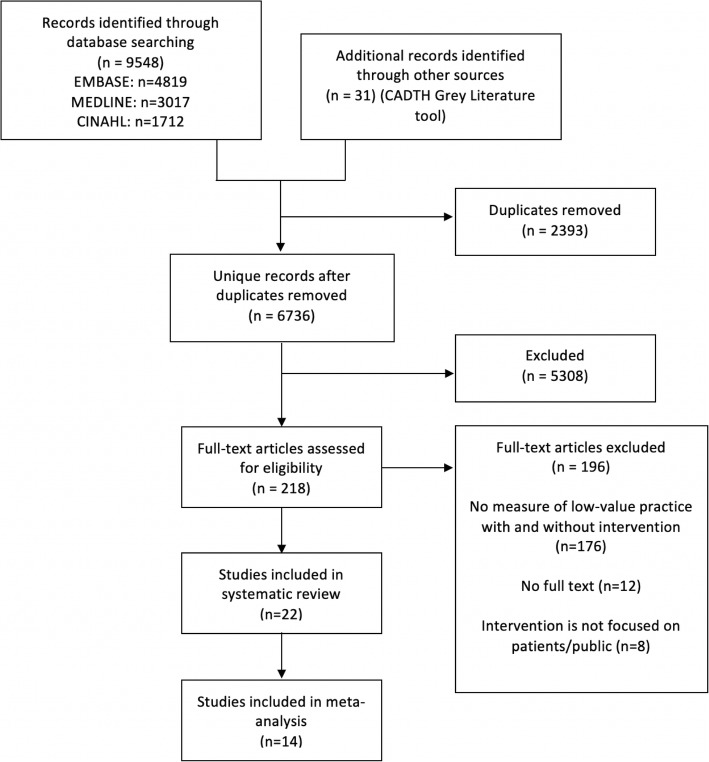
Selection of articles included in the review

Database and gray literature searches yielded 6736 unique citations, from which we reviewed 218 in full text and included 22 in the systematic review. The most common reason for exclusion after full-text review was the lack of reported use of the low-value practice with and without the intervention (Fig. 1). Of the 22 included studies, 14 reported the change in use of the low-value practice as the proportion of patients who received the low-value practice with and without the intervention, and were included in the meta-analysis.

### Study characteristics

Characteristics of included studies are described in Table 3. Most studies originated from the USA (*n* = 13, 59%), UK (*n* = 4, 18%), and Canada (*n* = 2, 9%). Nine studies were RCTs, and 13 studies were quasi-experimental. The most common type of low-value care was a medical treatment (*n* = 17, 77%). Studies commonly took place in primary care (*n* = 10, 45%), hospital inpatient wards (*n* = 6, 27%), or emergency departments (*n* = 3, 14%). Six studies (27%) focused on pediatric patients and their caregivers, four studies (18%) targeted adult patients, one study (5%) targeted geriatric patients, and 11 studies did not specify a target age group (50%). Studies used one or more of the following strategies for patient engagement: patient-oriented educational materials (*n* = 18, 82%), shared decision-making (*n* = 5, 23%), and media campaigns (*n* = 4, 18%). Three studies (14%) involved patients or the public in the development of intervention materials (e.g., shared decision-making tool, waiting room posters).

**Table 3:** Characteristics of the included studies (*n* = 22)

Author (year)	Country	Design	Number of patients included	Clinical setting	Low-value practice	Intervention	Control	Use of low-value practice with intervention	Use of low-value practice without intervention	Change in use of the low-value practice
**Randomized clinical trials**										
Macfarlane (2002) [28]	UK	Nested	212	Primary care	Antibiotics for acute bronchitis	Information leaflet on the natural course of lower respiratory symptoms and the advantage and disadvantages of antibiotics	General practitioner provided prompt card for informing patients that there is no indication for antibiotics	Proportion of patients who used antibiotics, 47%	Proportion of patients who used antibiotics, 62%	RR 0.76; 95% CI (0.59–0.97)
Francis (2009) [29]	UK	Cluster	558	Primary care	Antibiotic prescribing	Interactive booklet on respiratory tract infections	Usual care	Proportion of patients prescribed antibiotics, 19.5%	Proportion of patients prescribed antibiotics, 40.8%	RR 0.48; 95% CI (0.36, 0.64)
Legare (2012) [30]	Canada	Cluster	181	Primary care	Antibiotics for acute respiratory tract infections	Shared decision-making between patient and physicians	Usual care	Patients who used antibiotics following consultation, 27.2%	Patients who used antibiotics following consultation, 52.2%	RR 0.53; 95% CI (0.40, 0.70)
Tannenbaum (2014) [31]	Canada	Cluster	303	Community pharmacy	Benzodiazepines for older adults	Booklet providing information about risks, a tapering protocol, and prompt to discuss with physician	Usual care	Proportion of patients who discontinued benzodiazepine use, 27.0%	Proportion of patients who discontinued benzodiazepine use, 4.5%	RR 0.76; 95% CI (0.69, 0.85)
Schneiderman (2003) [32]	USA	Multi-center	551	Hospital	Non-beneficial life-sustaining treatments in the intensive care unit	Ethics consultations	Usual care	Mean (SD) days receiving ventilation, 6.52 (8.52)	Mean (SD) days receiving ventilation, 8.22 (11.16)	Absolute difference, 1.7 days; *P* = 0.03
Montgomery (2007) [33]	UK	Three-armed	742	Hospital	Non-indicated cesarean section	Information program: Information about probabilities of clinical outcomes Decision analysis: Decision tree recommended a “preferred option” based on women’s preferences and values	Usual care	Proportion of elective cesarean sections: information program, 49%; decision analysis, 41%	Proportion of elective cesarean sections, 50%	Information program: RR 0.95; 95% CI (0.79, 1.14) Decision analysis: RR 0.80; 95% CI (0.66, 0.98)
Hess (2012) [34]	USA	Two-armed, parallel	204	Emergency department	Cardiac stress testing in patients at low risk for acute coronary syndrome	Decision aid to improve patient knowledge and engage in shared decision-making	Usual care	Proportion of patients who received stress testing within 30 days of ED visit, 75%	Proportion of patients who received stress testing within 30 days of ED visit, 91%	RR 0.81; 95% CI (0.72, 0.93)
Hess (2016) [35]	USA	Multi-center	898	Emergency department	Cardiac stress testing in patients at low risk for acute coronary syndrome	Inform patients about their risk and options for care; shared decision aid	Usual care	Proportion of patients who received stress testing within 30 days of ED visit, 38.1%	Proportion of patients who received stress testing within 30 days of ED visit, 45.6%	RR 0.84; 95% CI (0.72, 0.98)
Navaee (2015)	Iran	Two-armed, parallel	67	Hospital	Cesarean section in primiparous women	Role-playing session about the advantages and disadvantages of cesarean section	Usual care	Proportion of patients who had a c-section, 2.9%	Proportion of patients who had a c-section, 15.6%	RR 0.18; 95% CI (0.02–1.48)
**Non-randomized intervention studies**										
Wheeler (2001) [36]	USA	Prospective observational study	144	Primary care	Antibiotic overuse	Educational videotape and written materials	No control group	Proportion of patients receiving antibiotic prescriptions, 4.2%	Proportion of patients receiving antibiotic prescriptions, 6.8%	RR 0.62; 95% CI (0.15, 2.49)
Perz (2002) [37]	USA	Before-and-after*	NR	Primary care	Antibiotic overuse	Educational materials for parents of young children and the general public	Usual care	Percent change in antibiotic prescription rate from baseline per 100 person-years, − 19%	Percent change in antibiotic prescription rate from baseline per 100 person-years, − 8%	Intervention-attributable decline, 11%; 95% CI (8–14%)
Dollman (2005) [38]	Australia	Before-and-after	~ 20,000	Primary care	Antibiotic use for upper respiratory tract infections, sinusitis, and otitis media	Pamphlets highlighting risks and benefits distributed to general practices and within the community	No control group	Defined daily dosages per 1000 population per day, 52.9	Defined daily dosages per 1000 population per day, 77.1	Overall reduction, 32%; *p* < 0.01
Gonzales (2005) [39]	USA	Non-randomized controlled trial*	1144	Primary care	Antibiotics for children with pharyngitis, antibiotics for adults with acute bronchitis	Educational materials mailed to households and available in physicians’ offices	Usual care	Proportion of patients prescribed antibiotics: adult, 36%; child, 30%	Proportion of patients prescribed antibiotics: adult, 60%; child, 34%	Adult: RR 0.60; 95% CI (0.51, 0.70) Child: RR 0.88; 95% CI (0.65, 1.18)
Ashe (2006) [40]	USA	Before-and-after	720	Primary care	Antibiotic overuse	Educational waiting room poster in physicians’ offices	Historical controls; usual care	Proportion of patients treated with antibiotics, 48.3%	Proportion of patients treated with antibiotics, 44.3%	RR 1.09; 95% CI (0.91, 1.31)
Gonzales (2008) [41]	USA	Non-randomized controlled trial	992	Primary care	Antibiotic overuse	Media campaign	Counties that did not receive a media campaign	Net antibiotic prescriptions per 1000 persons compared to comparison community (12 months post-intervention), − 5	Net antibiotic prescriptions per 1000 persons compared to control community (10 months pre-intervention), −1	8.8% net decrease in managed care-associated antibiotic dispenses per 1000 members; *P* = 0.03
Hemo (2009) [42]	Israel	Prospective observational study*	84,979	Not specified	Antibiotic use for upper respiratory tract infection, otitis media, pharyngitis	Media campaign	No control group	Post-campaign period vs. baseline: URI OR 0.749 (0.694, 0.808); otitis media OR 0.652 (0.591, 0.718); pharyngitis OR 0.931 (0.890, 0.973)	Pre-campaign period vs. baseline: URI OR 0.962 (0.891, 1.039); otitis media OR 0.970 (0.874, 1.076); pharyngitis OR 0.968 (0.929, 1.009)	
Morgan (2002) [43]	UK	Before-and-after	242	Primary care	Long-term use of benzodiazepines	Patient letter explaining risks, encouraging a reduction in intake, and prompt to contact physician for discussion	No control group	Mean defined daily dosages/patient, 283.0	Mean defined daily dosages/patient, 336.6	Absolute difference, 53.6; *P* < 0.001
Simpson (2010) [44]	USA	Before-and-after	531	Hospital	Elective labor induction	Educational classes on elective induction risk	No educational classes	Proportion of patients who received elective induction, 27.9%	Proportion of patients who received elective induction, 37%	RR 0.75; 95% CI (0.58, 0.96)
Engineer (2018) [45]	USA	Quality improvement project	176	Emergency department	Computed tomography for mild head injury	Electronic tool that involved a structured discussion between providers and caregivers	No control group	Proportion of patients who received head CT, 22.0%	Baseline head CT utilization in the pediatric ED population, 62.7%	RR 0.35; 95% CI (0.26, 0.47)
Arterburn (2006)	USA	Before-and-after	9515	Hospital	Unnecessary surgery for knee and hip osteoarthritis	Patient decision aids in DVD, website, and booklet format	No control group	Total hip replacement per 180 person-days, 0.34 Total knee replacement per 180 person-days, 0.09	Total hip replacement per 180 person-days, 0.46 Total knee replacement per 180 person-days, 0.16	Hip replacement relative rate, 0.74, *P* < 0.01 Knee replacement relative rate, 0.62, *P* < 0.01
Jerardi (2013)	USA	Quality improvement project	224	Hospital	Voiding cystourethrogram (VCUG) in children with first UTI with normal renal and bladder ultrasound (RBUS)	Educational materials and information sheets for patients and families	No control group	Proportion of patients with normal RBUS who received VCUG: 25.4%	Proportion of patients with normal RBUS who received VCUG: 84.9%	RR 0.29; 95% CI (0.17, 0.51)
Pugel (2018)	USA	Quality improvement project		Hospital	Complete blood counts (CBCs), electrocardiograms (EKGs) as routine screening tests in physical examination visits, age-inappropriate dual-energy absorptiometry (DEXA) scans, imaging for uncomplicated headache	Patient-targeted materials produced by Consumer Reports, including exam room posters, patient education materials in waiting areas and exam rooms	No control group	CBCs, 3.16%; EKGs, 0.33%; DEXA scans, 2.02%; imaging for uncomplicated headache, 6.88%	CBCs, 42.7%; EKGs, 15.9%; DEXA scans, 25.4%; imaging for uncomplicated headache, 10.8%	Absolute difference (95% CI): CBCs 39.54% (39.0–40.0); EKGs 15.57% (15.1–15.8); DEXA scans 23.38% (22.5–24.5); imaging for uncomplicated headache 3.92% (3.3–4.6)

*CT* computed tomography, *RR* risk ratio, *ED* emergency department, *SD* standard deviation, *URI* upper respiratory infection

*Adequately adjusted for confounding

### The effect of de-implementation interventions that engage patients on the use of low-value care

Of the 22 included studies, 19 (86%) reported a statistically significant decrease in the use of the targeted low-value practice (Table 3). Low-value treatments were targeted in 17 interventions (77%), of which 12 targeted low-value medications. All but one of these interventions included a patient-targeted education component; six interventions used patient-targeted education exclusively [28, 31, 36, 39, 40, 43], one intervention supplemented patient-targeted education with shared decision-making [29], and two interventions used both patient-targeted education and media campaigns [37, 38]. One intervention engaged patients solely through shared decision-making [30]. All but two [36, 40] of these interventions targeting low-value medication reported decreases in the proportion of patients using the medication, ranging from 15 to 25%. However, in one intervention, medication use was reduced in adults (absolute difference 24%, *P* < 0.02), but not in children (absolute difference 4%, *P* = 0.18) [39]. Media campaigns were the sole patient-engagement strategy in two interventions [41, 42], both of which reported a significant reduction in low-value medication use. Further details on the strategies used in these interventions are reported in Table 3.

Targeted low-value procedures included non-indicated cesarean sections [33, 46], elective labor induction [44], unnecessary surgery for knee and hip osteoarthritis [47], and non-beneficial life-sustaining treatments in the intensive care unit (Table 3) [32]. One study aiming to reduce non-indicated cesarean sections designed two interventions, an informational program and a guided decision analysis, of which only the guided decision analysis was effective [33]. Elective labor inductions and non-beneficial life-sustaining treatments were successfully reduced through educational classes and ethics consultations, respectively. Unnecessary surgery for hip and knee osteoarthritis was successfully reduced through the use of patient education and decision aids.

Low-value diagnostic tests were targeted in five interventions which reduced use by 7.5–40.7% [34, 35, 45, 48, 49]; one study reduced the use of computed tomography (CT) scans for mild head injury in children [45], one study reduced voiding cystourethrograms for children with normal renal and bladder ultrasounds [49], two studies reduced cardiac stress testing in adults at low risk for acute coronary syndrome, and one study reduced the use of screening tests commonly ordered in physical examination visits such as complete blood counts (CBCs) and electrocardiograms (EKGs) [34, 35, 48]. Of these five interventions, three studies took place within an emergency department and used shared decision-making. Four of the studies included educational materials to inform patients about risks and care options [34, 35].

**Fig. 2:**
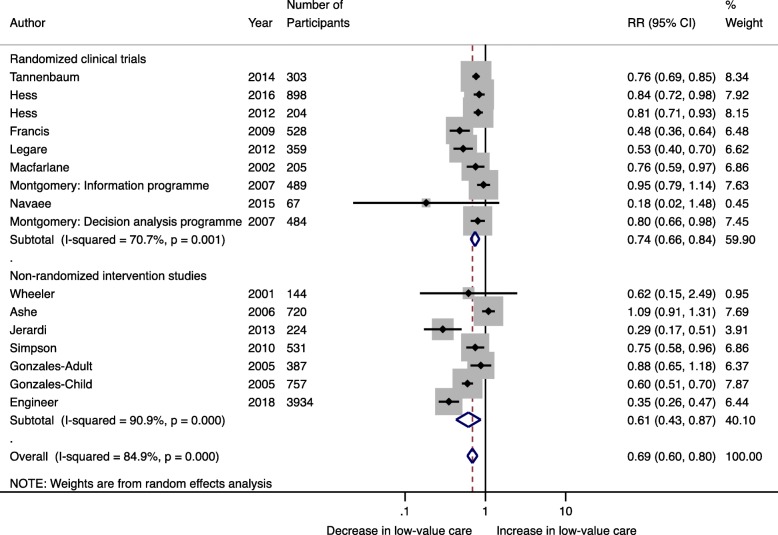
Random effects meta-analysis stratified by study design examining the effect of de-implementation interventions that engage patients within the patient-clinician interaction on the use of low-value care

**Fig. 3:**
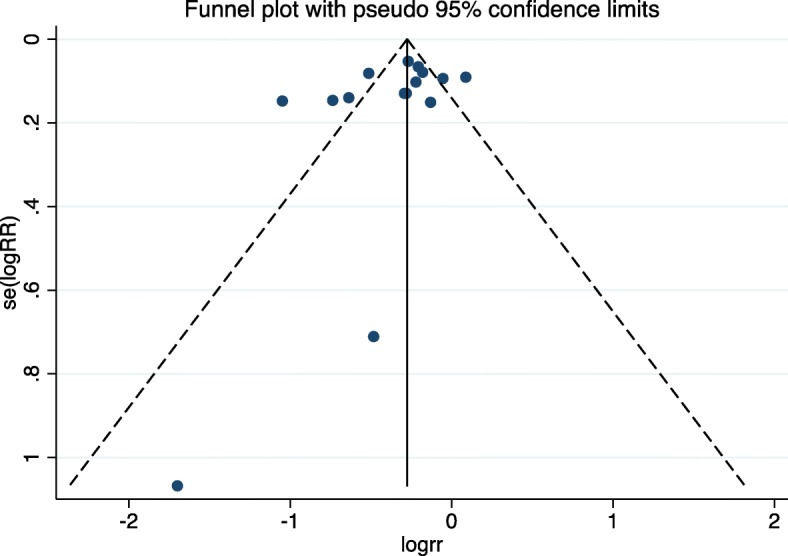
Funnel plot with pseudo 95% CIs

Fourteen studies inclusive of 10,234 patients were included in the meta-analysis. Pooling data indicated that de-implementation interventions that engage patients within the patient-clinician interaction decreased the use of low-value care by 31% (RR 0.69; 95% CI 0.60–0.80; *I*^2^ 84.9%) (Fig. 2). This effect was similar in RCTs (*n* = 8 studies, 3537 patients) (RR 0.74; 95% CI 0.66–0.84; *I*^2^ 70.7%) and quasi-experimental studies (*n* = 6 studies, 6697 patients) (RR 0.61; 95% CI 0.43–0.87; *I*^2^ 90.9%) (Fig. 2). Egger’s test (*p* = 0.201) and assessment of the funnel plot (Fig. 3) for asymmetry indicated a lack of publication bias.

### Quality assessment

#### Randomized clinical trials

Of eight RCTs, five had an overall low risk of bias [29–31, 34, 35], three had an overall unclear risk of bias [28, 32, 33], and one had an overall high risk of bias (Additional File 1). All but two studies had a low risk of selection bias, and all studies had a low risk of attrition bias. The reporting bias domain was unclear in three studies.

#### Quasi-experimental studies

All quasi-experimental studies clearly reported objectives and interventions; however, potentially confounding variables were poorly reported. Nine studies (69%) aimed to recruit participants that were representative of the source population, and 12 (92%) used staff, places, and facilities that were representative of treatment received by most patients. None of the studies provided sufficient information to determine whether participants were representative of the population from which they were recruited. None of the studies blinded participants or outcome assessors. The median quality score was 57 (IQR 52–66). Five studies above the median were classified as “higher” quality, six studies below the median were classified as “lower” quality, and two studies that were equivalent to the median were classified as “average” quality (Additional File 2).

### Exploration for sources of heterogeneity

**Fig. 4:**
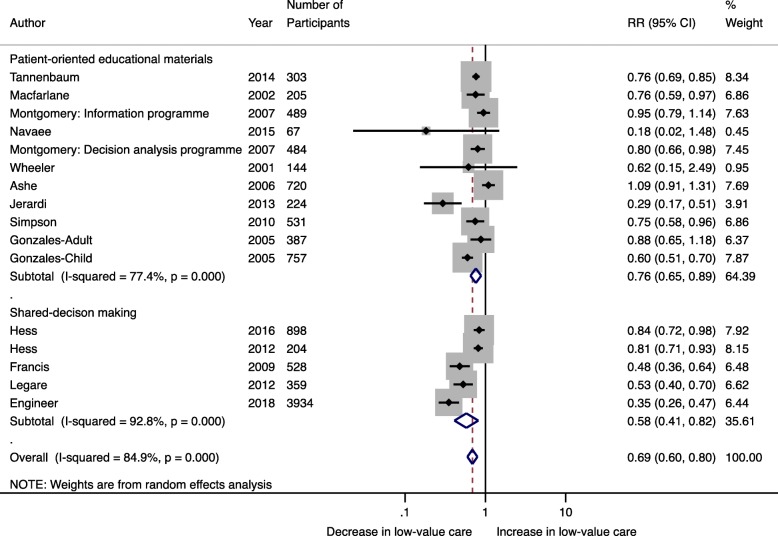
Random effects meta-analysis of the association between patient-targeted interventions to reduce low-value care by the strategy for patient involvement

**Fig. 5:**
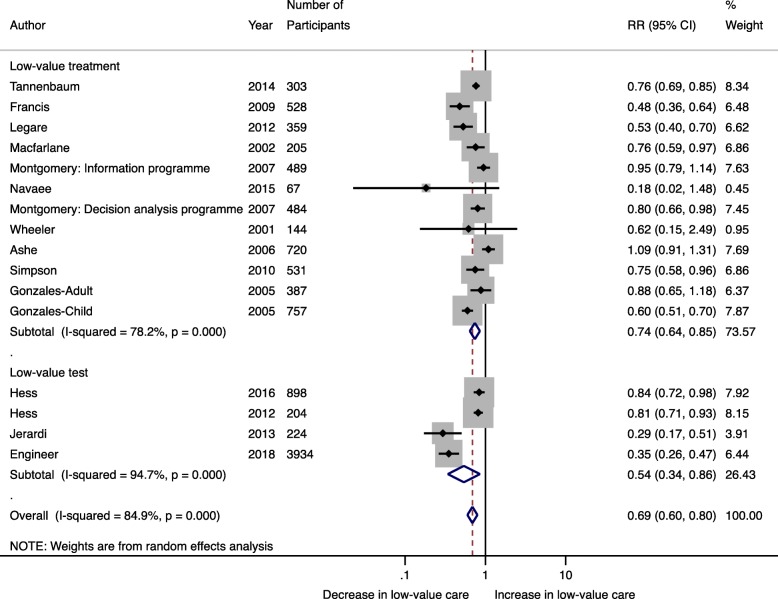
Stratified by type of low-value care, random effects meta-analysis examining the effect of de-implementation interventions that engage patients within the patient-clinician interaction on the use of low-value care

**Fig. 6:**
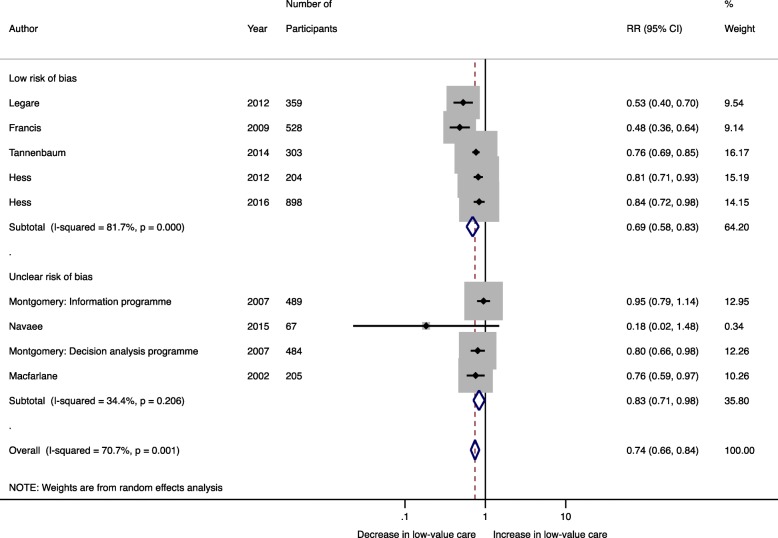
Among RCTs stratified by summary risk of bias assessment, random effects meta-analysis examining the effect of de-implementation interventions that engage patients within the patient-clinician interaction on the use of low-value care

Stratified analyses were conducted to explore heterogeneity. Among the five RCTs with low risk of bias, studies used diverse strategies for public engagement and various low-value practices: four studies used shared decision-making, whereas one used patient-targeted educational materials, and three studies targeted medications, whereas two targeted diagnostic tests. Stratification by the strategy for patient engagement suggested that shared decision-making had a greater effect on reducing the use of low-value care (RR 0.58; 95% CI 0.41–0.82; *I*^2^ 92.8%) in comparison (meta-regression *P* = 0.07) to patient-oriented educational materials (RR 0.76; 95% CI 0.65–0.89; *I*^2^ 77.4%) (Fig. 4). Similar effect sizes were found when the meta-analysis was stratified by studies targeting low-value tests (*n* = 4) (RR 0.54; 95% CI 0.34–0.86; *I*^2^ 94.7%) and low-value treatments (*n* = 10) (RR 0.74; 95% CI 0.64–0.85; *I*^2^ 78.2%) (Fig. 5). When the meta-analysis was restricted to RCTs with low risk of bias (*n* = 5), the effect of patient-targeted interventions remained significant (RR 0.69; 95% CI 0.58–0.83) although there was still a high degree of heterogeneity (*I*^2^ 81.7%; Q-statistic *P* < 0.001) (Fig. 6). Meta-regression, although limited by the small number of studies, suggested that neither variability due to the targeted low-value care (test vs. treatment) nor strategy for patient engagement (shared decision-making vs. patient-oriented educational materials) contributed to inter-study heterogeneity (Additional File 3).

## Discussion

We identified nine RCTs and thirteen quasi-experimental studies that prospectively evaluated the effect of de-implementation interventions that engaged patients within the patient-clinician interaction on the use of low-value care. Compared to a prior knowledge synthesis examining this topic [17], we identified additional relevant studies and provide a tangible, quantified estimate of the effect of these interventions. Patient engagement occurred through patient-oriented educational materials, mass media campaigns, and shared decision-making between the patient and clinician. Studies examined a broad array of low-value care including potentially unnecessary tests (e.g., computed tomography scans for mild head injuries), treatments (e.g., antibiotics for upper respiratory tract infections), and surgeries (e.g., cesarean sections). The most common clinical context was adult patients making decisions about medical treatments in primary care. Meta-analysis demonstrated that patient engagement within the patient-clinician interaction reduced the use of low-value care by an average of 31% (range 20 to 40%). Although this effect estimate was limited by inter-study heterogeneity, it is the first to quantify the potential impact of such de-implementation interventions, and despite the heterogeneity, effects remained consistent when examined in subgroups defined by different strategies for patient engagement, types of low-value care, and study design. Researchers, policymakers, and decision-makers should consider the patient-targeted intervention as a strategy for reducing low-value care.

Our results add to and compare favorably with prior research examining a similar question [17]. Colla et al. performed a systematic review examining interventions to reduce low-value care, inclusive of 108 articles, of which 19 reported on interventions that involved a patient education component. Narrative synthesis among the 19 articles concluded that patient-oriented education is an effective strategy for patient engagement within the patient-clinician interaction and can successfully reduce low-value care. However, many of their studies used patient-targeted strategies within multicomponent interventions that included clinician-targeted strategies (e.g., clinical decision support, provider feedback). This makes it challenging to understand which components of the intervention (i.e., patient-targeted or clinician-targeted) were actually effective and whether there is an advantage to focusing on one group or another. Although it would seem logical that the synergistic effects achieved from simultaneously engaging patients and clinicians would be greater than those from strategies engaging the two parties separately, this has yet to be adequately examined. Understanding the effects of patient-targeted interventions is important because clinicians have indicated that important barriers to reducing low-value care include patient care expectations and the risk of patient dissatisfaction if expectations are not met [15, 16]. Moreover, given the costs and resources associated with multicomponent de-implementation initiatives, understanding which strategies have the greatest effect on reducing low-value care is important for advancing the science of de-implementation and informing how to best reduce low-value care. Patient-targeted interventions within the patient-clinician interaction may help to mitigate these challenges. As clinician-targeted strategies did not meet inclusion criteria for our systematic review, this enabled us to isolate studies that only reported on patient-targeted interventions within the patient-clinician interaction and examine their effect on use of low-value care.

Acknowledging the potential benefits of patient engagement in reducing low-value care, patient engagement in clinical decision-making is often viewed as challenging, time-consuming, and potentially costly [50, 51]. Our study demonstrates that the ensuant reductions in low-value care make tackling these challenges worthwhile. However, it is important to consider that the nature and success of patient-targeted de-implementation interventions likely depend on patient and clinician characteristics, clinical context, and the targeted low-value practice. For example, one study found that patient-oriented educational materials reduced unnecessary medication use among adult, but not pediatric, patients [39]. This finding is congruent with and potentially explained by evidence from recent systematic reviews indicating that informational components may be sufficient for interventions that target patients (e.g., adults), but that caregivers (e.g., parents of children) require supplemental information that enables activation (i.e., prompting action) and/or collaboration (i.e., engagement with clinicians or others) [52, 53]. Because these de-implementation interventions occur within the patient-clinician interaction, their success will also depend on the characteristics of participating clinicians and their ability and intention to engage the patient in strategies like shared decision-making. Interventions may benefit from a training component wherein clinicians learn how to effectively engage patients through the selected strategy. In addition, most interventions in our review targeted low-value practices that lacked efficacy in a primary care setting by providing patients with educational materials. This approach to patient engagement was evidently effective for a low-value practice associated with minimal risk for patients with low illness burden. However, for low-value practices provided to patients with greater illness burden cared for in clinical contexts associated with greater risk (e.g., emergency departments, hospital inpatient wards, etc.), de-implementation interventions that seek to engage patients and/or their caregivers will likely require more interaction and collaboration with clinicians. Although few studies in our review examined patient-focused interventions in high acuity contexts, one study did successfully implement an intervention that involved families and clinicians in ethics consultations to discuss non-beneficial life-sustaining treatments in intensive care units [32]. Future research should examine the effect of patient engagement on the use of low-value care in acute care contexts.

### Strengths and limitations

A notable strength of this study is the rigorous methodology which included a peer-reviewed search strategy and adherence to published guidelines regarding systematic review and meta-analysis methodology. In addition, our review was narrow in scope and focused solely on de-implementation interventions that engaged patients within the patient-clinician interaction, which is arguably one of the most critical clinical contexts for influencing the use of low-value care. Our meta-analysis quantified the effect of patient engagement within the patient-clinician interaction and confirmed its effectiveness as an approach to reducing low-value care, while highlighting important heterogeneity within published literature. However, this study must be interpreted within the context of its limitations. First, the English language restriction may have omitted relevant articles, yet it is unlikely that this would have altered our main findings. Second, the main outcome of the proportion of patients who received the low-value practice may have overestimated the use of certain low-value practices. For example, if a study’s outcome was the number of patients given a prescription for benzodiazepines and some patients did not end up using the prescription, the true number would have been overestimated. However, this misclassification bias is expected to be non-differential and would suggest that if anything, our results are a conservative estimate of the true population effect. Third, this study focused on low-value clinical interventions, and therefore, we cannot comment on the de-implementation of non-medical forms of care. In addition, the majority of included studies targeted medication prescribing in primary care. This limits the generalizability of our results. Further research is needed to determine if the observed effects persist in other clinical contexts (e.g., emergency department). Fourth, there was significant inter-study heterogeneity that affects interpretation of the pooled estimates. Given the nature of our research question, we anticipated observing inter-study heterogeneity within our pooled estimates. We proceeded with calculating pooled effect estimates in spite of this because (1) effect estimates from individual studies, especially those that were of higher quality, were similar; (2) pooled estimates have greater utility relative to individual study effect estimates, as they may be used to facilitate comparisons with meta-analyses of other types of interventions aiming to reduce low-value care (e.g., provider-targeted interventions); (3) collectively, this small group of studies represents the totality of available evidence for evaluating the average impact of patient-targeted interventions on use of low-value care, so restricting our scope to a specific low-value practice, patient population, or patient-engagement tool would have resulted in a small number of studies precluding any meaningful findings; and (4) similar approaches have been employed in other meta-analyses investigating the utility of interventions that span clinician disciplines and patient populations, such as decision aids [54]. Finally, although this review indicated that de-implementation interventions that engage patients within the patient-clinician interaction is a promising approach for reducing low-value care, we must consider that the implementation and effectiveness of these interventions will be significantly influenced by clinicians, given that they hold the authority to order a low-value test or treatment. This review did not aim to explore how clinicians may respond to or support patient engagement in de-implementation interventions, and therefore, further research is required to determine how to appropriately support and engage clinicians within such interventions.

## Conclusions

This systematic review with meta-analysis suggests that de-implementation interventions that engage patients within the patient-clinician interaction through patient-targeted educational materials or shared decision-making are effective in decreasing the use of low-value care, especially for medical treatments prescribed within primary care. Additional research should seek to understand the utility of patient-targeted interventions in the acute care context and how the effectiveness of patient-targeted interventions compares to that of clinician-targeted or multicomponent interventions. However, based on the results of this study, de-implementation interventions that seek to reduce low-value medical treatments provided to patients in a primary care setting should incorporate patient engagement using tailored educational and shared decision-making tools.

## Supplementary information

**Additional file 1. **Risk of bias and quality assessment of included randomized clinical trials (*n* = 9).

**Additional file 2. **Quality assessment for non-randomized interventions using the Downs & Black tool (*n* = 13).

**Additional file 3.** Results from meta-regression analysis.

## Data Availability

All data generated or analyzed during this study are included in this published article and its supplementary information files.

## Abbreviations

CADTH: Canadian Agency for Drugs and Technologies in Health
PRESS: Peer Review of Electronic Search Strategies
PRISMA: Preferred Reporting Items for Systematic Reviews and Meta-analyses
RCT: Randomized clinical trials

## References

[1] Elshaug AG, Rosenthal MB, Lavis JN (2017). Levers for addressing medical underuse and overuse: achieving high-value health care. Lancet.

[2] Shrank WH, Rogstad TL, Parekh N. Waste in the US health care system. JAMA. 2019. 10.1001/jama.2019.13978. 10.1001/jama.2019.13978 31589283

[3] Badgery-Parker T, Pearson S-A, Dunn S (2019). Measuring hospital-acquired complications associated with low-value care. JAMA Intern Med.

[4] Korenstein D, Keyhani S, Troy A (2018). Development of a conceptual map of negative consequences for patients of overuse of medical tests and treatments. JAMA Intern Med.

[5] Bece A, Hamilton C, Hickey BE (2013). Over 150 potentially low-value health care practices: an Australian study. Med J Aust.

[6] Prasad V, Vandross A, Toomey C (2013). A decade of reversal: an analysis of 146 contradicted medical practices. Mayo Clin Proc.

[7] Cassel CK, Guest JA (2012). Choosing wisely. Jama.

[8] Garner S, Littlejohns P (2011). Disinvestment from low value clinical interventions: NICEly done?. BMJ.

[9] Canadian Institute for Health Information. Unnecessary care in Canada. Ottawa: CIHI; 2017.

[10] Rosenberg A, Agiro A, Gottlieb M (2015). Early trends among seven recommendations from the choosing wisely campaign. JAMA Intern Med.

[11] Niven DJ, Rubenfeld GD, Kramer AA (2015). Effect of published scientific evidence on glycemic control in adult intensive care units. JAMA Intern Med.

[12] Van Bodegom-Vos L, Davidoff F, Marang-Van De Mheen PJ. Implementation and de-implementation: two sides of the same coin? . 10.1136/bmjqs-2016-005473. 10.1136/bmjqs-2016-005473 27512102

[13] Colla CH (2014). Swimming against the current — what might work to reduce low-value care?. N Engl J Med.

[14] Brownlee S, Berman A (2016). Defining value in health care resource utilization: articulating the role of the patient.

[15] Buist D (2015). Primary care clinicians’ perspectives on reducing low-value care in an integrated delivery system. Perm J.

[16] Zikmund-Fisher BJ, Kullgren JT, Fagerlin A (2017). Perceived barriers to implementing individual choosing wisely® recommendations in two national surveys of primary care providers. J Gen Intern Med.

[17] Colla CH, Mainor AJ, Hargreaves C, et al. Interventions aimed at reducing use of low-value health services: a systematic review. 2017. 10.1177/1077558716656970. 10.1177/1077558716656970 27402662

[18] Sypes EE, de Grood C, Clement FM, Parsons Leigh J, Whalen-Browne L, Stelfox HT, Niven DJ. Understanding the public's role in reducing low-value care: a scoping review. Implementation Science. In press. (accepted March 23 2020). 10.1186/s13012-020-00986-0 PMC7137456 32264926

[19] Institute JB (2011). Joanna Briggs Institute reviewers’ manual.

[20] Moher D, Liberati A, Tetzlaff J, *et al.* Preferred reporting items for systematic reviews and meta-analyses: the PRISMA statement David Moher and colleagues introduce PRISMA, an update of the QUOROM guidelines for reporting systematic reviews and meta-analyses , for the PRISMA Group. doi:10.1136/bmj.b2535.

[21] Canadian Agency for Drugs and Technologies in Health. Grey matters: a practical tool for searching health-related grey literature. 2015.https://www.cadth.ca/resources/finding-evidence/grey-matters. Accessed 11 Apr 2018).

[22] McGowan J, Sampson M, Salzwedel DM (2016). PRESS peer review of electronic search strategies: 2015 guideline statement. J Clin Epidemiol.

[23] Landis JR, Koch GG (1977). The measurement of observer agreement for categorical data. Biometrics.

[24] Higgins JPT, Altman DG, Gotzsche PC (2011). The Cochrane Collaboration’s tool for assessing risk of bias in randomised trials. BMJ.

[25] Downs SH, Black N (1998). The feasibility of creating a checklist for the assessment of the methodological quality both of randomised and non-randomised studies of health care interventions.

[26] DerSimonian R, Laird N (1986). Meta-analysis in clinical trials. Control Clin Trials.

[27] Higgins JPT, Thompson SG, Deeks JJ (2003). Measuring inconsistency in meta-analyses. BMJ.

[28] Macfarlane J, Holmes W, Gard P (2002). Primary care patient information leaflet. Bmj.

[29] Francis NA, Butler CC, Hood K (2009). Effect of using an interactive booklet about childhood respiratory tract infections in primary care consultations on reconsulting and antibiotic prescribing: a cluster randomised controlled trial. BMJ.

[30] Légaré F, Labrecque M, Cauchon M (2012). Training family physicians in shared decision-making to reduce the overuse of antibiotics in acute respiratory infections: a cluster randomized trial. CMAJ.

[31] Tannenbaum C, Martin P, Tamblyn R (2014). Reduction of inappropriate benzodiazepine prescriptions among older adults through direct patient education: the EMPOWER cluster randomized trial. JAMA Intern Med.

[32] Schneiderman LJ, Gilmer T, Teetzel HD (2003). Effect of ethics consultations on nonbeneficial life-sustaining treatments in the intensive care setting. JAMA.

[33] Montgomery AA, Emmett CL, Fahey T (2007). Two decision aids for mode of delivery among women with previous caesarean section: randomised controlled trial. BMJ.

[34] Hess EP, Knoedler MA, Shah ND (2012). The chest pain choice decision aid: a randomized trial. Circ Cardiovasc Qual Outcomes.

[35] Hess EP, Hollander JE, Schaffer JT, et al. Shared decision making in patients with low risk chest pain: prospective randomized pragmatic trial. BMJ. 2016;355. 10.1136/bmj.i6165. 10.1136/bmj.i6165 PMC5152707 27919865

[36] Wheeler JG, Fair M, Simpson PM (2001). Impact of a waiting room videotape message on parent attitudes toward pediatric antibiotic use. Pediatrics.

[37] Perz JF, Craig AS, Coffey CS (2002). Changes in antibiotic prescribing for children after a community-wide campaign. JAMA.

[38] Dollman WB, LeBlanc VT, Stevens L (2005). A community-based intervention to reduce antibiotic use for upper respiratory tract infections in regional South Australia. Med J Aust.

[39] Gonzales R, Corbett KK, Leeman-Castillo BA (2005). The “minimizing antibiotic resistance in Colorado” project: impact of patient education in improving antibiotic use in private office practices. Health Serv Res.

[40] Ashe D, Patrick PA, Stempel MM, et al. Educational posters to reduce antibiotic use. J Pediatr Heal Care. 2006;20:192–7. 10.1016/j.pedhc.2005.12.017. 10.1016/j.pedhc.2005.12.017 16675380

[41] Gonzales R, Corbett KK, Wong S, et al. Get smart Colorado. Medical Care. 2008;46:597–605. 10.1097/MLR.0b013e3181653d2e 18520314

[42] Hemo B, Shamir-Shtein NH, Silverman BG (2009). Can a nationwide media campaign affect antibiotic use?. Am J Manag Care.

[43] Morgan JD, Wright DJ, Chrystyn H (2002). Pharmacoeconomic evaluation of a patient education letter aimed at reducing long-term prescribing of benzodiazepines. Pharm World Sci.

[44] Simpson KR, Newman G, Chirino OR (2010). Patient education to reduce elective labor inductions. MCN, Am J Matern Nurs.

[45] Engineer RS, Podolsky SR, Fertel BS (2018). A pilot study to reduce computed tomography utilization for pediatric mild head injury in the emergency department using a clinical decision support tool and a structured parent discussion tool. Pediatr Emerg Care.

[46] Navaee M, Abedian Z (2019). Effect of role play education on primiparous women’s fear of natural delivery and their decision on the mode of delivery. Iran J Nurs Midwifery Res.

[47] Arterburn D, Wellman R, Westbrook E (2012). Introducing decision aids at group health was linked to sharply lower hip and knee surgery rates and costs. Health Aff.

[48] Pugel S, Stallworth JL, Pugh LB (2018). Choosing wisely in Georgia: a quality improvement initiative in 25 adult ambulatory medicine offices. Jt Comm J Qual Patient Saf.

[49] Jerardi KE, Elkeeb D, Weiser J (2013). Rapid implementation of evidence-based guidelines for imaging after first urinary tract infection. Pediatrics.

[50] Kovacs Burns K, Bellows M, Eigenseher C (2014). ‘Practical’ resources to support patient and family engagement in healthcare decisions: a scoping review. BMC Health Serv Res.

[51] Domecq JP, Prutsky G, Elraiyah T (2014). Patient engagement in research: a systematic review. BMC Health Serv Res.

[52] Fiest KM, McIntosh CJ, Demiantschuk D (2018). Translating evidence to patient care through caregivers: a systematic review of caregiver-mediated interventions. BMC Med.

[53] Gagliardi AR, Légaré F, Brouwers MC (2015). Patient-mediated knowledge translation (PKT) interventions for clinical encounters: a systematic review. Implement Sci.

[54] Stacey D, Légaré F, Lewis K (2017). Decision aids for people facing health treatment or screening decisions. Cochrane Database Syst Rev.

