# Supplementary material for: Engaging patients in de-implementation interventions to reduce low-value clinical care: a systematic review and meta-analysis
Source: BMC Med. 2020 May 8;18:116. doi: 10.1186/s12916-020-01567-0 (PMC7206676; doi:10.1186/s12916-020-01567-0)
Supplement: Supplementary file 1 — Additional file 1. Risk of bias and quality assessment of included randomized clinical trials (n = 9). [file 12916_2020_1567_MOESM1_ESM.docx]

Additional File 1. Risk of bias and quality assessment of included randomized clinical trials

| **Study**  **(year)** | **Selection bias (Random sequence generation)** | **Selection bias (Allocation concealment)** | **Performance bias (Blinding of participants and personnel)** | **Detection bias (Blinding of outcome assessment)** | **Attrition bias (Incomplete outcome data)** | **Reporting bias (Selective reporting)** | **Other bias** | **Summary assessment** |
| --- | --- | --- | --- | --- | --- | --- | --- | --- |
| Hess  (2012)^1^ | Low risk of bias | Low risk of bias | High risk of bias | Low risk of bias | Low risk of bias | Low risk of bias | Low risk of bias | Low risk of bias |
| Macfarlane (2002)^2^ | Unclear risk of bias | Low risk of bias | Low risk of bias | Low risk of bias | Low risk of bias | Unclear risk of bias | Low risk of bias | Unclear risk of bias |
| Tannenbaum (2014)^3^ | Low risk of bias | Low risk of bias | Low risk of bias | Low risk of bias | Low risk of bias | Low risk of bias | Low risk of bias | Low risk of bias |
| Francis  (2009)^4^ | Low risk of bias | Low risk of bias | Low risk of bias | Low risk of bias | Low risk of bias | Low risk of bias | Low risk of bias | Low risk of bias |
| Schneiderman (2003)^5^ | Low risk of bias | Low risk of bias | Low risk of bias | Low risk of bias | Low risk of bias | Unclear risk of bias | Low risk of bias | Unclear risk of bias |
| Legare  (2012)^6^ | Low risk of bias | Low risk of bias | High risk of bias | Low risk of bias | Low risk of bias | Low risk of bias | Low risk of bias | Low risk of bias |
| Montgomery (2007)^7^ | Low risk of bias | Low risk of bias | High risk of bias | Unclear risk of bias | Low risk of bias | Low risk of bias | Low risk of bias | Unclear risk of bias |
| Hess  (2016)^8^ | Low risk of bias | Low risk of bias | High risk of bias | Low risk of bias | Low risk of bias | Low risk of bias | Low risk of bias | Low risk of bias |
| Navaee  (2015)^9^ | High risk of bias | Unclear risk of bias | High risk of bias | Unclear risk of bias | Low risk of bias | Unclear risk of bias | High risk of bias | High risk of bias |
