# Supplementary material for: Engaging patients in de-implementation interventions to reduce low-value clinical care: a systematic review and meta-analysis
Source: BMC Med. 2020 May 8;18:116. doi: 10.1186/s12916-020-01567-0 (PMC7206676; doi:10.1186/s12916-020-01567-0)
Supplement: Supplementary file 2 — Additional file 2. Quality assessment for non-randomized interventions using the Downs & Black tool (n = 13). [file 12916_2020_1567_MOESM2_ESM.docx]

| **Checklist Item** | **Engineer^9^** | **Hemo^10^** | **Morgan^11^** | **Ashe^12^** | **Dollman^13^** | **Wheeler^14^** | **Perz^15^** | **Simpson^16^** | **Gonzales^17^** | **Gonzales^18^** | **Pugel^19^** | **Arterburn^20^** | **Jerardi^21^** |
| --- | --- | --- | --- | --- | --- | --- | --- | --- | --- | --- | --- | --- | --- |
| Q1 | 1 | 1 | 1 | 1 | 1 | 1 | 1 | 1 | 1 | 1 | 1 | 1 | 1 |
| Q2 | 1 | 1 | 1 | 1 | 1 | 1 | 1 | 1 | 0 | 1 | 1 | 1 | 1 |
| Q3 | 1 | 1 | 1 | 1 | 0 | 1 | 1 | 1 | 1 | 0 | 0 | 1 | 0 |
| Q4 | 1 | 1 | 1 | 1 | 1 | 1 | 1 | 1 | 1 | 1 | 1 | 1 | 1 |
| Q5 | 0 | 1 | 0 | 0 | 0 | 1 | 2 | 0 | 1 | 0 | 0 | 2 | 0 |
| Q6 | 1 | 0 | 1 | 1 | 1 | 1 | 1 | 1 | 0 | 1 | 1 | 0 | 1 |
| Q7 | 1 | 1 | 1 | 0 | 0 | 1 | 1 | 0 | 1 | 0 | 1 | 1 | 1 |
| Q8 | 0 | 0 | 0 | 0 | 0 | 0 | 0 | 0 | 1 | 0 | 0 | 0 | 0 |
| Q9 | n/a | n/a | n/a | n/a | n/a | n/a | n/a | 0 | 0 | 1 | n/a | n/a | n/a |
| Q10 | 0 | 1 | 0 | 1 | 1 | 0 | 1 | 1 | 1 | 1 | 0 | 1 | 1 |
| Q11 | UTD | 1 | 1 | 1 | 1 | 1 | 1 | 0 | 0 | UTD | 1 | 1 | 1 |
| Q12 | UTD | UTD | UTD | UTD | UTD | UTD | UTD | UTD | UTD | UTD | UTD | UTD | UTD |
| Q13 | 1 | 1 | 1 | 1 | 1 | 1 | 1 | 0 | 1 | 1 | 1 | 1 | 1 |
| Q14 | 0 | 0 | 0 | 0 | 0 | 0 | 0 | 0 | 0 | 0 | 0 | 0 | 0 |
| Q15 | 0 | 0 | 0 | 0 | 0 | 0 | 0 | 0 | 0 | 0 | 0 | 0 | 0 |
| Q16 | 1 | 1 | 1 | 1 | 1 | 1 | 1 | 1 | 1 | 1 | 1 | 1 | 1 |
| Q17 | 1 | 1 | 1 | 1 | 1 | 1 | 1 | UTD | 1 | UTD | 1 | 1 | 1 |
| Q18 | 1 | 1 | 1 | 1 | 0 | 1 | 1 | 1 | 1 | 1 | 1 | 1 | 1 |
| Q19 | 1 | 1 | 1 | 1 | 1 | 0 | UTD | UTD | 0 | UTD | UTD | UTD | UTD |
| Q20 | 1 | 1 | 0 | 1 | 0 | 1 | 1 | 1 | 1 | 1 | 1 | 1 | 1 |
| Q21 | 0 | 1 | 1 | 1 | 1 | 1 | 1 | 1 | 1 | 0 | 1 | 1 | 1 |
| Q22 | n/a | n/a | n/a | n/a | n/a | n/a | 1 | UTD | 1 | 1 | n/a | 0 | n/a |
| Q25 | 0 | 1 | 0 | 0 | 0 | UTD | 1 | 0 | 0 | 1 | 0 | 1 | 0 |
| Q26 | UTD | 1 | UTD | 0 | UTD | 0 | UTD | UTD | UTD | 1 | UTD | UTD | UTD |
| Q27 | 0 | 0 | 0 | 1 | 0 | 1 | 0 | 1 | 0 | 1 | 0 | 0 | 0 |
| **Total** | 12/23 | 17/23 | 13/23 | 15/23 | 11/23 | 15/23 | 18/24 | 11/25 | 14/25 | 13/25 | 12/23 | 16/24 | 13/23 |
| **% Score** | 52% | 74% | 57% | 65% | 48% | 65% | 75% | 44% | 56% | 52% | 52% | 67% | 57% |
| **Overall quality** | Lower | Higher | Average | Higher | Lower | Higher | Higher | Lower | Lower | Lower | Lower | Higher | Average |

**Additional File 2. Quality assessment for non-randomized interventions using the Downs & Black tool (n=13)**

For Q5: 0=No, 1=Partially, 2=Yes and for all other Qs: 0=No, 1=Yes; UTD: Unable to determine; n/a: not applicable
