# Supplementary material for: Engaging patients in de-implementation interventions to reduce low-value clinical care: a systematic review and meta-analysis
Source: BMC Med. 2020 May 8;18:116. doi: 10.1186/s12916-020-01567-0 (PMC7206676; doi:10.1186/s12916-020-01567-0)
Supplement: Supplementary file 3 — Additional file 3. Results from meta-regression analysis. [file 12916_2020_1567_MOESM3_ESM.docx]

Additional File 3. Results from meta-regression analysis

| **Variable** | **Coefficient of logRR(s.e.)** | **Adjusted R^2^** | ***P* value** |
| --- | --- | --- | --- |
| Low-value practice | .134 (.198) | -7.11% | 0.510 |
| Strategy for patient engagement | .303 (.152) | 20.95% | 0.067 |
